# Development of bioluminescent Group B streptococcal strains for longitudinal infection studies

**DOI:** 10.1038/s41598-024-74346-z

**Published:** 2024-10-18

**Authors:** Inês Lorga, Rafaela Geraldo, Joana Soares, Liliana Oliveira, Arnaud Firon, Elva Bonifácio Andrade

**Affiliations:** 1 ICBAS - Instituto de Ciências Biomédicas de Abel Salazar, Universidade do Porto, Porto, Portugal; 2 i3S - Instituto de Investigação e Inovação em Saúde, Universidade do Porto, Porto, Portugal; 3 CBQF - Centro de Biotecnologia e Química Fina, Escola Superior de Biotecnologia da Universidade Católica Portuguesa, Porto, Portugal; 4 Microbiology Department, Institut Pasteur, Université Paris-Cité, Paris, France; 5 E2S - Escola Superior de Saúde, Instituto Politécnico do Porto, Porto, Portugal

**Keywords:** Bacteria, Infectious diseases, Experimental models of disease, Paediatric research

## Abstract

Group B *Streptococcus* (GBS) remains the leading bacterial cause of invasive neonatal disease, resulting in substantial morbidity and mortality. New therapeutic approaches beyond antibacterial treatment to prevent neonatal disease outcomes are urgent. One significant limitation in studying GBS disease and progression is the lack of non-invasive technologies for longitudinal studies. Here, we develop and compare three bioluminescent GBS strains for *in vivo* pathogenic analysis. Bioluminescence is based on the *luxABCDE* operon on a replicative vector (*lux*GBS-CC17), and the red-shifted firefly luciferase on a replicative vector (*ffluc*GBS-CC17) or integrated in the genome (*gluc*GBS-CC17). We show that *lux*GBS-CC17 is suitable for *in vitro* analysis but does not produce a significant bioluminescent signal in infected pups. In contrast, the *ffluc*GBS-CC17 results in a strong bioluminescent signal proportional to the organ colonisation level. However, the stability of the replicative vector depends on the route of infection, especially when pups acquire the bacteria from infected vaginal mucosa. Stable chromosomal integration of luciferase in *gluc*GBS-CC17 leads to significant bioluminescence in both haematological and vertical infection models associated with high systemic colonisation. These strains will allow the preclinical evaluation of treatment efficacy against GBS invasive disease using whole-mouse bioluminescence imaging.

**Supplementary Information:**

The online version contains supplementary material available at 10.1038/s41598-024-74346-z.

## Introduction

Group B *Streptococcus* (GBS) remains the leading bacterial pathogen responsible for invasive disease and death in infants under two months old^1^. GBS colonises the lower genital tract of pregnant women and can be transmitted vertically to the newborn *in utero* or during birth, leading to early-onset disease (EOD), mostly associated with sepsis and pneumonia, or late-onset disease (LOD), frequently presented as meningitis^2^. Invasive GBS disease results in a mortality rate of 10–15% and life-long neurodevelopment impairment (NDI) in up to 50% of survivors^3^. GBS meningitis is particularly concerning, as its incidence has been increasing over the last decade^4–6^. Moreover, no neuroprotective treatment is currently available, and *intrapartum* antibiotic prophylaxis is ineffective to prevent LOD meningitis^7^, highlighting the necessity for developing new preventive and therapeutic solutions.

Over the past decades, significant efforts have been made to elucidate the pathophysiology and long-term NDI resulting from GBS neonatal disease, leading to the establishment of several animal models, including hematogenous, oral and direct brain infections^8–11^. Recently, our group developed an animal mouse model that mimics the natural human route of infection, *i.e.* mother-to-progeny transmission, through intra-vaginal colonisation of pregnant females^12^. However, these models still do not provide a complete understanding of the disease establishment and progression^2,12^. Traditional colony-forming unit (CFU) counting methods have limitations, including the need to euthanise animals to assess organ colonisation^13^. Using non-invasive bioluminescence imaging (BLI) would enable longitudinal *in vivo* studies, enhancing our understanding of the pathophysiology of GBS neonatal disease. Additionally, BLI would facilitate testing the efficacy of novel prophylactic and therapeutic treatments. Moreover, this technique aligns with the ethical principles of the 3Rs in animal experiments by reducing and refining animal usage, allowing each animal to be studied across multiple time points and organs, rather than sacrificing one animal per time point for limited organ studies^13,14^.

Bioluminescent reporter systems have been developed in several pathogenic streptococcal species^15–18^. In GBS, luminescence has been obtained with a replicative vector containing the luciferase gene (*luc*) from the firefly *Photinus poyralis*^15^. The firefly luciferase converts D-luciferin, its substrate, to oxyluciferin, emitting light with a maximum intensity of 560 nm^19^. The replicative vector is stabilised by antibiotic selection and a toxin-antitoxin (TA) system plasmid stability for up to 10 days *in vitro* even without the antibiotic selective pressure^15^. However, this system was not evaluated *in vivo*, in animal models of infection. Group A *Streptococcus* (GAS) has been the most studied streptococcal species with bioluminescent reporters^15–18^. Initially, the bacterial luciferin-luciferase operon (*luxABCDE*) was used, which has the advantage of not requiring an external luciferin substrate for bioluminescent light emission (maximum intensity at 490 nm)^18^. Nevertheless, this system produces a weaker signal, and the vector is less stable^18^. To improve sensitivity *in vivo*, a variant using the red-shifted firefly luciferase (*ffluc*), which emits light at 610 nm, was created in GAS^18^. Bioluminescent streptococcal strains have been used for studying disease progression, antimicrobial compounds, vaccine efficacy or even pathogenic synergism^20–23^, highlighting the importance and wide-range applicability of BLI for enhanced *in vivo* studies.

In the present study, we aimed to develop bioluminescent GBS strains to further improve our knowledge of neonatal GBS invasive disease. We found that vector-based approaches are unstable and inefficient for longitudinal studies with GBS, even with a stable *ffluc* plasmid carrying a TA system. However, genomic insertion of the red-shifted firefly luciferase gene into GBS creates a stable bioluminescent strain suitable for evaluating severe GBS disease using whole-mouse live imaging.

## Results

### Development and assessment of a *lux* bioluminescent reporter system in GBS

To generate a GBS bioluminescent reporter strain, we used the BM110 strain, of capsular serotype III and belonging to the CC-17 hypervirulent lineage responsible for more than 80% of meningitis cases^12,24,25^. We first tested an available bioluminescent vector-based system containing the *luxABCDE* operon, which encodes the luciferase and its aldehyde substrate, thereby eliminating the need for an exogenous substrate^13,26^. To this end, we introduced the replicative vector pSL101P_32_, containing the constitutively active P32 promotor^27,28^ to generate the GBS strain *lux*GBS-CC17.

**Fig. 1:**
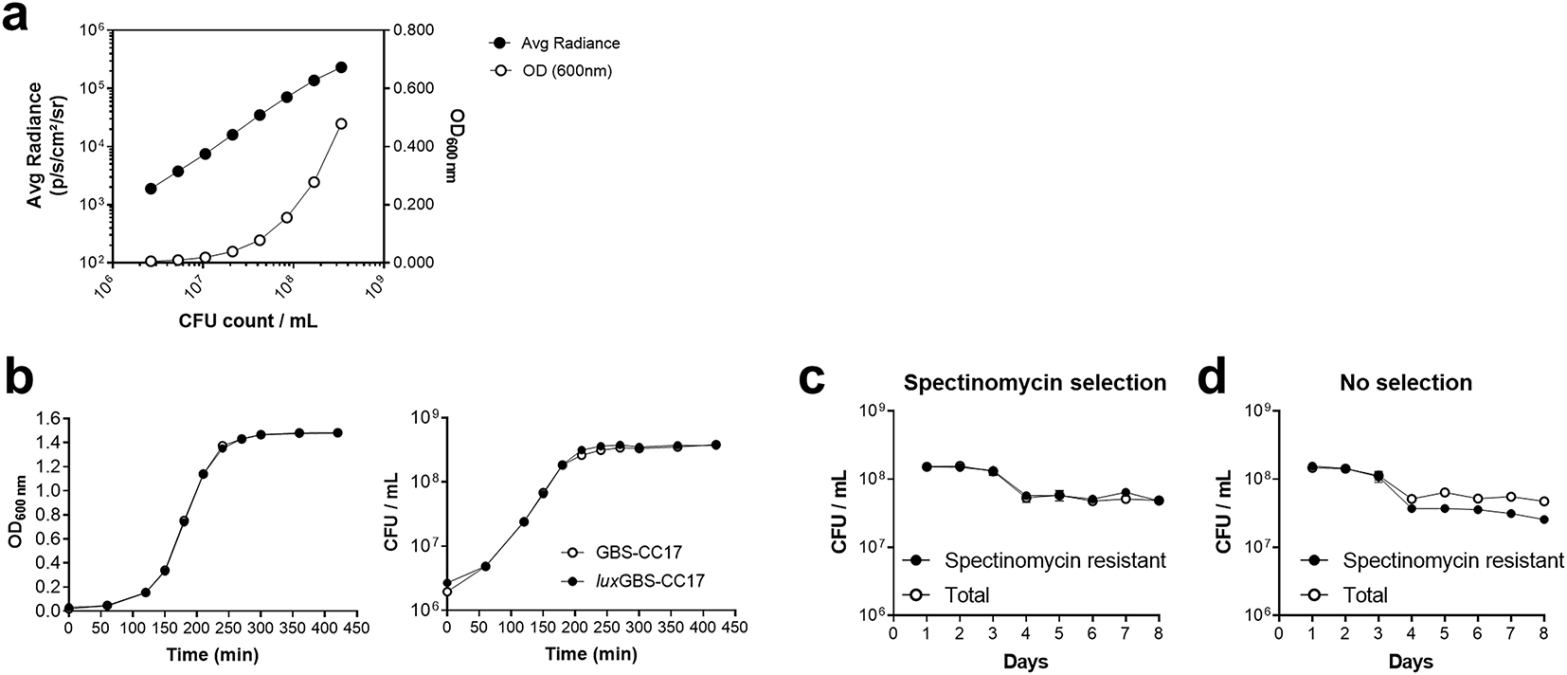
*In vitro* bioluminescence with the pSL101P_32_ replicative vector in GBS. The pSL101P_32_ vector was introduced in the BM110 wild-type strain (WT) to give origin to the strain *lux*GBS-CC17. **(a)** Correlation of bioluminescent signal, optical density (OD) and CFU count. *lux*GBS-CC17 was grown until the mid-log phase and serially diluted in 96-well plate. Bioluminescence, OD and CFU quantified. Field symbols represent radiance, whereas open symbols OD. Bioluminescent signal plotted as radiance (photons/seconds/cm^2^/sr). Results represent data from 3 independent experiments. **(b)** Growth curves of WT and *lux*GBS-CC-17 strains. Optical density (left) measured every 30–60 min for 7 h and the corresponding GBS numbers (right). Mean values ± SEM data pooled from 3 independent experiments. Comparisons were performed by two-way ANOVA. **(c-d)** The *lux*GBS-CC-17 strain was daily diluted 1:1000 into fresh TH medium (with or without spectinomycin) and grown for 24 h, for 8 consecutive days. **(c)** CFU enumeration of both strains plated on TH medium with or **(d)** without selection for CFU count. Mean values ± SEM data pooled from 3 independent experiments. Comparisons were performed by two-way ANOVA.

To evaluate whether light emission could be expressed as a function of cell growth, bacteria from the mid-log phase were serially diluted, and the luminescence (emission OD_490nm_), optical density (OD_600nm_), and cell counts (CFU/mL) were determined. The bioluminescent signal of *lux*GBS-CC17 positively correlated with both OD_600nm_ and the number of CFU, indicating that the bioluminescent signal reliably predicts bacterial count (Fig. 1a). Moreover, the pSL101P_32_ vector does not have a fitness cost since the growth curve was equivalent to the one of wild-type (WT) GBS (GBS-CC17) under standard conditions in TH (Fig. 1b). To assess the stability of the pSL101P_32_ vector, we grew serial cultures of *lux*GBS-CC17 with or without the selective antibiotic (spectinomycin) for 8 days. Bacteria were quantified each 24 h, by evaluating CFU on selective and non-selective media (total *versus* spectinomycin resistant bacteria). No significant differences were found between total and spectinomycin resistant GBS strains, even when grown without selective pressure, indicating that the plasmid was stable *in vitro* (Fig. 1c and d).

**Fig. 2:**
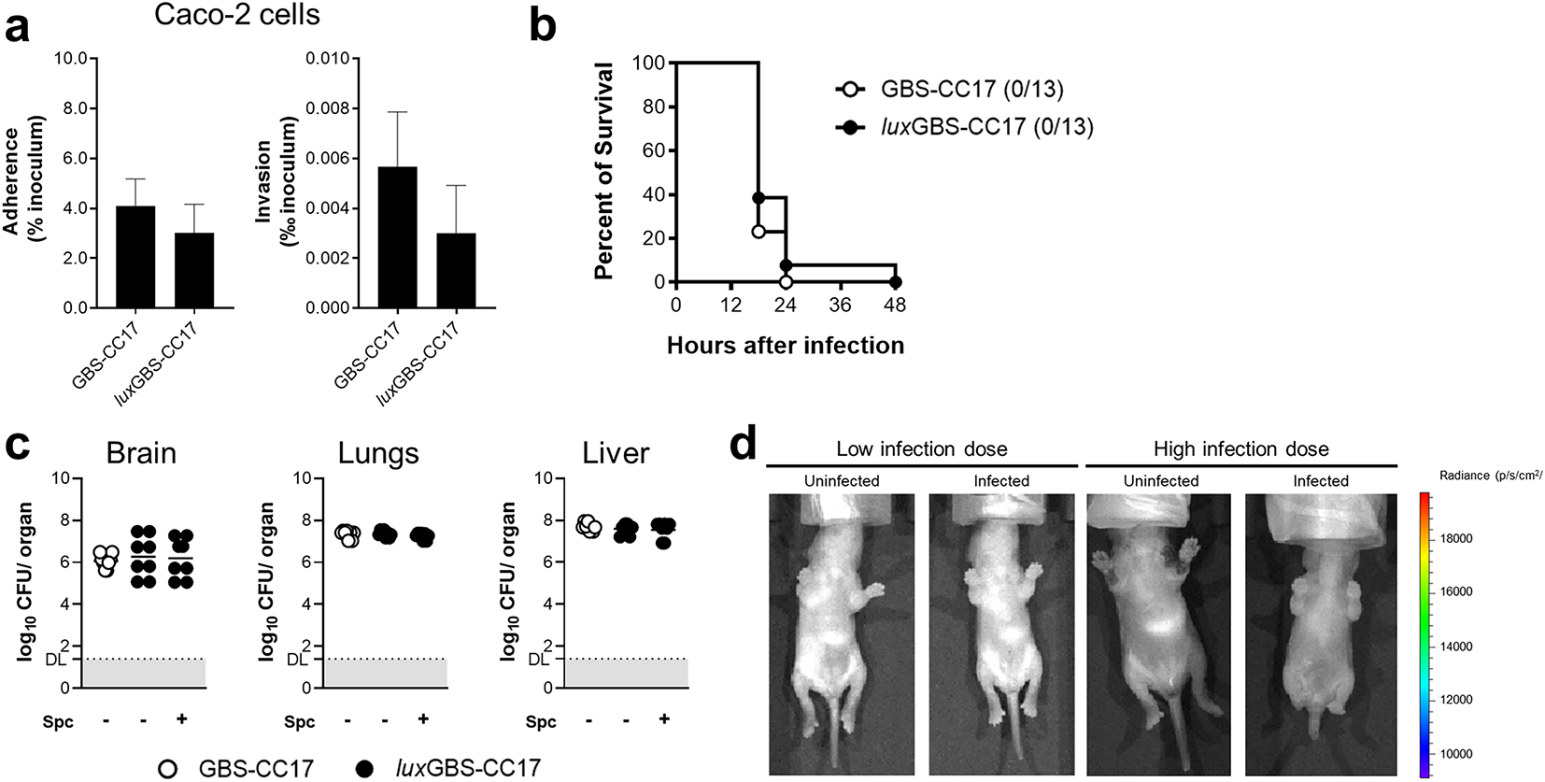
Infectivity and *in vivo* bioluminescence of *lux*GBS-CC-17.**(a)** Caco-2 cells were infected with WT or *lux*GBS-CC17 strains at a multiplicity of infection of 30, for 3 h, in triplicates. Bacteria adhesion and invasion were determined. Results are expressed relative to the initial inoculum. Data are shown as mean + SEM pool from 3 independent experiments. **(b-c)** Neonatal mice were inoculated intraperitoneally with 5 × 10^4^ CFU of WT or *lux*GBS-CC17. **(b)** Kaplan-Meier survival curve monitored for 48 h. Numbers in parenthesis indicate the number of animals that survived versus the total number of infected animals (*n* = 13). Results represent data pooled from 2 independent experiments. Comparisons with the log-rank Mantel-Cox test. **(c)** Bacterial load in the brain, lungs and liver 18 h post-infection (hpi). The organs from WT infected pups were plated on TH medium, while those from *ffluc*GBS-CC17 were plated on TH with and without spectinomycin. Data are shown as means, and each symbol represents data from individual pups (*n* = 8). DL, detection limit = 1.4 (25 CFU). **(d)** Neonatal mice were inoculated intraperitoneally with 5 × 10^4^ (low dose) or 1 × 10^5^ (high dose) CFU of *lux*GBS-CC17 and euthanised 18 hpi. Representative images of uninfected or infected pups resulting from the superimposition of the bioluminescence signal map and a grey-scale photograph of the mice in the ventral position.

To evaluate the infectivity of the bioluminescent strain, we tested its adhesion and invasion properties on human Caco-2 cells. No significant differences were observed between *lux*GBS-CC17 and WT GBS-infected cells for their ability to adhere to and invade the Caco-2 enterocytes (Fig. 2a). To test the virulence of *lux*GBS-CC17, newborn mice at postnatal day (P) 2 were intraperitoneally (i.p.) infected with 5 × 10^5^ CFU of *lux*GBS-CC17 or GBS-CC17. No differences were observed in the survival rates of the pups between groups (Fig. 2b). To investigate invasion and colonisation of internal organs, we next determined the number of CFU in the brain, lungs, and liver 18 h post-infection (hpi). No differences were observed between groups, showing that the replicative vector did not affect bacterial virulence (Fig. 2c). Moreover, all *lux*GBS-CC17 colonies recovered from infected organs are spectinomycin-resistant, as assayed by CFU counting on media with and without antibiotics, indicating that the vector is stable for the duration of the experiment (Fig. 2c). To determine whether this strain generates a robust bioluminescent signal, mice infected with lower (5 × 10^4^ CFU) or higher (1 × 10^5^ CFU) doses of *lux*GBS-CC17 were imaged using an IVIS Lumina LT (Fig. 2d). Unexpectedly, as shown in Fig. 2d, no signal was detected on any tested pup regardless of the infection dose, suggesting that the individual luminescence signal was not high enough to be detected. Overall, these results show that the bioluminescent *luxABCDE*-based GBS strain is not efficient for *in vivo* studies.

### Development and evaluation of a firefly luciferase-expressing GBS strain for *in vivo* short-term infection studies

**Fig. 3:**
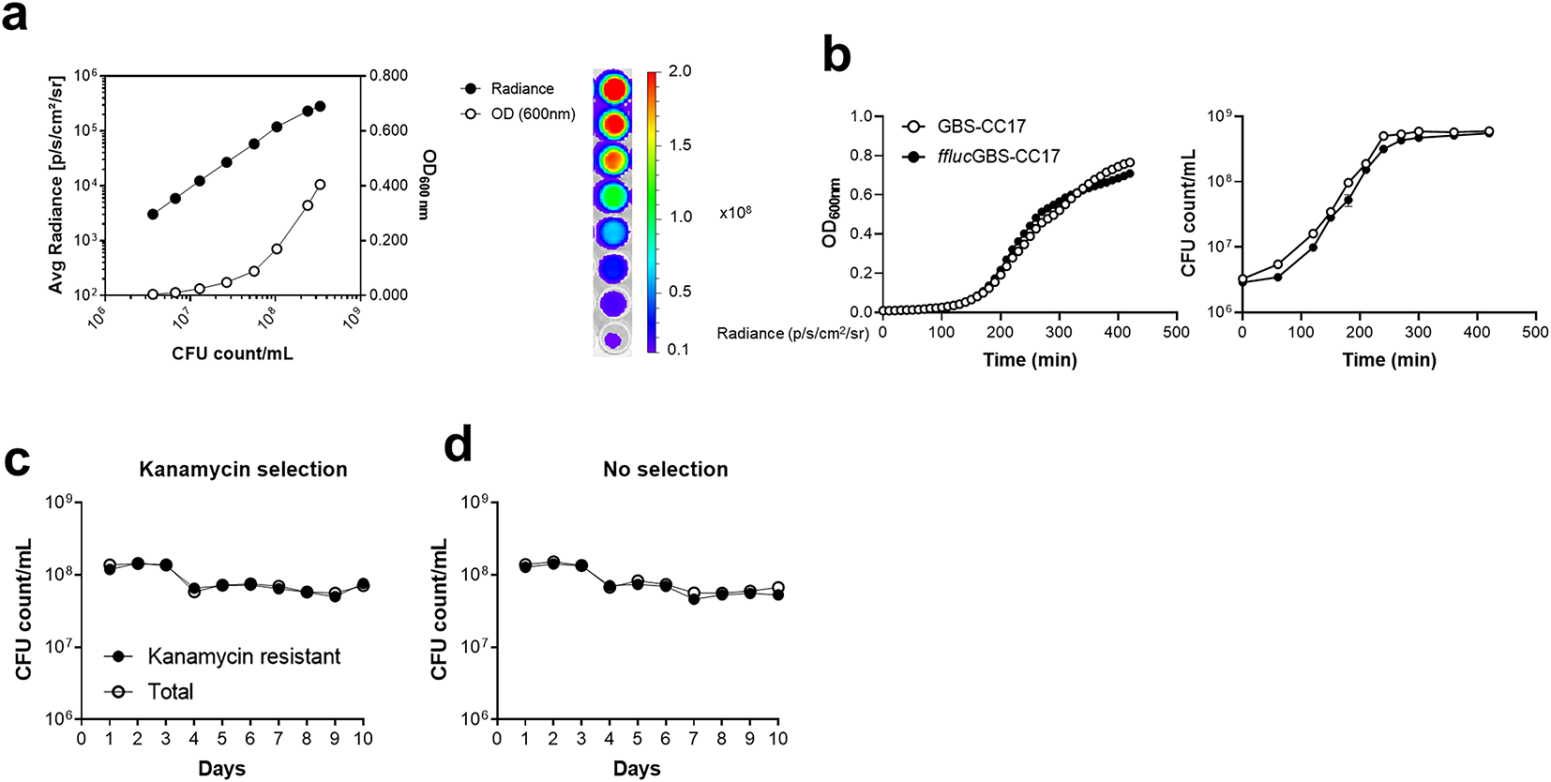
*In vitro* bioluminescence with the pLZ12Km2-P23R: TA: fflucRT replicative vector in GBS. The WT strain was transformed with the pLZ12Km2-P23R: TA: fflucRT vector to give the *ffluc*GBS-CC17 strain. **(a)** Correlation of bioluminescent signal, optical density (OD) and CFU count. The *ffluc*GBS-CC17 strain was grown until the mid-log phase and serially diluted on a 96-well plate. Left: Bioluminescence (Radiance, 0.5 mg/mL of Luciferin) and OD quantification on a plate reader (Synergy 2), and CFU counts. Field symbols represent radiance, whereas open symbols represent OD. Right: Bioluminescence signal imaged using IVIS Lumina LT. Areas with high photon emission correspond to the red colour and the areas with lower emission are displayed in blue. The detection of bioluminescent signals by the system resulted in the generation of signal maps overlaid on a grey-scale photograph of the 96-well plate. Bioluminescent signal plotted as radiance (photons/seconds/cm^2^/sr). Results represent data pooled from 3 independent experiments. **(b)** Growth curves of WT and *ffluc*GBS-CC17 strains. Optical density (left) measured every 10 min for 7 h on Synergy 2. GBS numbers were counted every 30–60 min (right). Mean values ± SEM data pooled from 3 independent experiments. Comparisons were performed by two-way ANOVA. **(c-d)** WT and *ffluc*GBS-CC17 strains were daily diluted 1:1000 into fresh TH medium (with or without kanamycin) and grown for 24 h, for 10 consecutive days. Numbers of kanamycin resistant GBS and total GBS cells grown in medium with **(c)** or without **(d)** kanamycin selection were counted. Mean values ± SEM data pooled from 3 independent experiments. Comparisons were performed by two-way ANOVA.

**Fig. 4:**
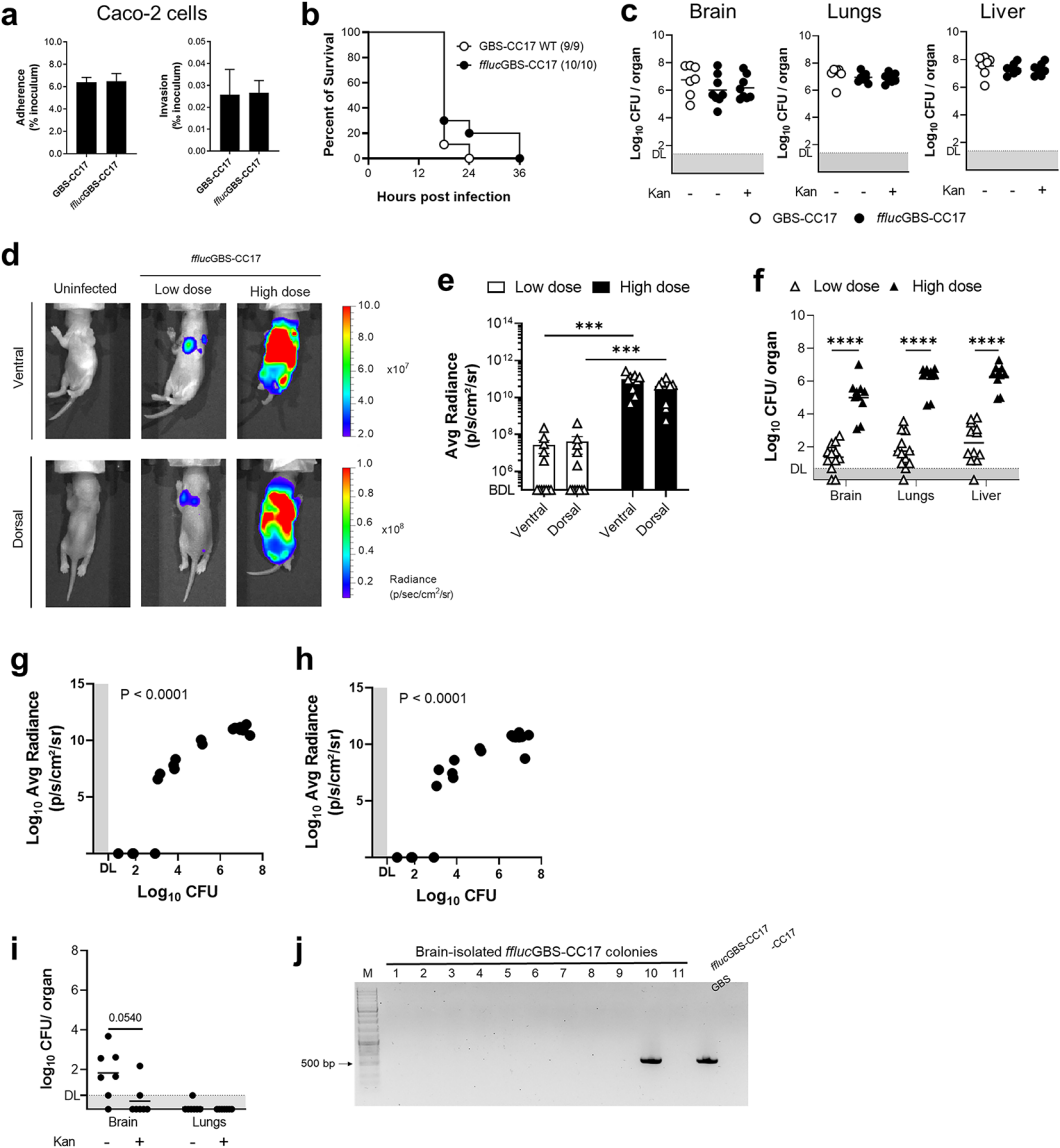
Infectivity and *in vivo* bioluminescence of *ffluc*GBS-CC17. **(a)** Caco-2 cells were infected with WT or *ffluc*GBS-CC17 for 3 h. Bacteria adhesion and invasion were determined. Results are expressed relative to the initial inoculum. Data shown as mean + SEM from 3 independent experiments. **(b-c)** Neonatal mice were inoculated intraperitoneally with 5 × 10^4^ CFU of WT or *ffluc*GBS-CC17. Data pooled from 3 independent experiments. **(b)** Kaplan-Meier survival curve monitored for 36 h. Numbers in parenthesis: survivors versus total (*n* = 9 WT; *n* = 10 *ffluc*GBS-CC17). Log-rank Mantel-Cox test comparisons. **(c)** Bacterial load in indicated organs at 18 h post-infection (hpi), plated on TH with or without kanamycin. Data shown as mean, each symbol represents one pup (*n* = 7 WT; *n* = 8 *ffluc*GBS-CC17). DL, detection limit = 1.4. **(d-h)** Pups were inoculated intraperitoneally with 5 × 10^4^ (low dose) or 1 × 10^5^ (high dose) CFU of *ffluc*GBS-ST17 and analysed 18 hpi. **(d)** Representative images by superimposition of the bioluminescence signal map and a grey-scale photograph. **(e)** The regions of interest (ROIs) were used to quantify the whole mouse bioluminescence signal and expressed as average radiance (photons/seconds/cm^2^/steradian). Mean ± SEM, each symbol represents one individual. BDL, below detection limit. **(f)** Bacterial load in the indicated organs. Data shown as mean, each symbol represents one animal (*n* = 12 Low dose; *n* = 10 High dose). Statistical analysis using unpaired Student’s t-test. *** *P* < 0.001; ****P < 0.0001. DL, detection limit = 0.7. **(g-h)** Correlation between colonisation and average radiance in infected pups in the **(g)** ventral and **(h)** dorsal positions. Each symbol represents one animal. DL, detection limit. *P < 0.0001. **(i-j)** Pregnant mice were intra-vaginally inoculated with 8 × 10^4^*ffluc*GBS-CC17. Three-day-old pups were analysed. **(i)** Bacterial load in the indicated organs. Data shown as mean pooled from 2 independent experiments. Each symbol represents one animal (*n* = 7). Statistical analysis by unpaired Student’s t-test. * *P* < 0.05. DL, detection limit = 0.7. **(j)** Representative agarose gel showing colony PCR for screening *ffluc*GBS-CC17 collected from pups’ brains, using plasmid-targeted primers (495 bp amplicon). M, molecular weight marker (10 kb GeneRuler DNA ladder mix); 1–11, bacteria isolates; *ffluc*GBS-CC17 and WT GBS-CC17 as controls.

Aiming at developing a bioluminescent GBS strain for longitudinal studies, we next used a replicative vector containing the red-shifted firefly luciferase, pLZ12Km2-P23R: TA: ffluc^15,18^ and the constitutively active P23 promotor^29^. The red-shifted firefly *ffluc* luciferase system has a peak of light emission with a longer wavelength (610 nm) which enhances tissue penetration compared to the *lux* reporter system^18,30^. Therefore, we introduced the pLZ12Km2-P23R: TA: ffluc vector in BM110 by electroporation, creating the *ffluc*GBS-CC17 strain. Similarly to *lux*GBS-CC17, the bioluminescent signal of *ffluc*GBS-CC17 positively correlated with the OD_600nm_ and the number of CFU (Fig. 3a). The presence of the plasmid did not affect bacterial growth, as indicated by overlapping growth curves of the WT strain and *ffluc*GBS-CC17 (Fig. 3b). Moreover, the plasmid remained stable *in vitro* for up to 10 days without antibiotic selective pressure (Fig. 3c and d). Using the human enterocytes cell line Caco-2, we confirmed that the *ffluc*GBS-CC17 strain adheres to and invades cells similarly to the WT strain (Fig. 4a). Moreover, when assessing the infectivity *in vivo* using a neonatal mouse model of infection, no differences in survival were observed between pups infected with *ffluc*GBS-CC17 or with the WT strain (Fig. 4b). Bacterial loads in the brain, lungs and liver were comparable between groups 18 hpi (Fig. 4c). Additionally, no differences were found when enumerating *ffluc*GBS-CC17 CFU in organs plated on selective and non-selective media, confirming plasmid stability *in vivo* (Fig. 4c).

To validate the strain for BLI, P2 pups were infected with a low (5 × 10^4^ CFU) and high (1 × 10^5^ CFU) doses of *ffluc*GBS-CC17. At 18 hpi, pups were administered D-Luciferin subcutaneously (s.c.) 5 minutes before BLI acquisition, and images were captured in both dorsal and ventral positions (Fig. 4d). As expected, high inoculum levels resulted in significantly (*P* = 0.0003) stronger bioluminescent signals, measured by average radiance (photons/second/cm^2^/steradian) (Fig. 4d-e). However, 7 out of 12 pups infected with the low dose did not exhibit bioluminescent signal (Fig. 4e). Thus, to determine whether these differences were correlated with different bacterial colonisation, GBS burden in the brain, lungs and liver were evaluated. Pups infected with the low dose presented significantly (*P* = 0.0003) lower bacterial loads than those infected with the high dose (Fig. 4f). Among the 7 pups without bioluminescent signal, only 2 had CFU counts below the detection level, suggesting that this inoculum led to colonisation below the detection threshold of the IVIS system. Importantly, a positive correlation was observed when plotting the whole-body radiance with the sum of bacterial colonisation in the lungs, liver, and brain (Fig. 4g and h). Taken together, these data show that the overall lack of bioluminescent signal intensity was due to reduced number of GBS in these organs.

To determine whether the bioluminescent *ffluc*GBS-CC17 strain could be used as a reliable bacterial quantification method in a mouse model mimicking the pathophysiology of GBS disease, newborn mice were infected vertically using our mouse model of infection^12^, adapted for C57BL/6 mice. Briefly, pregnant mice were colonised with GBS-CC17 (WT or *ffluc*) in their vaginal mucosa at gestational days 16 and 17. No selective antibiotic was given to the mother or their progeny. Three-day-old pups were given D-luciferin, anaesthetised, subjected to BLI, and sacrificed for organ colonisation assessment. Unexpectedly, no bioluminescent signal was detected in any of the tested pups (data not shown). As expected, the organ colonisation in the brain, lungs and liver is lower than in the systemic infection, as our infection model usually results in decreased bacterial load (Fig. 4c and i). Contrary to what was found in the i.p. model, CFU counts in the brain differed when plated on agar-medium with and without antibiotic selection (Fig. 4i). In the brain, only 1 out of 7 pups had detectable GBS CFU on kanamycin-containing medium, while 5 out of 7 pups had no detectable CFU when using non-selective medium (Fig. 4i). Regarding the lungs, only 1 pup presented colonisation without selection, and none with antibiotic selection (Fig. 4i). To investigate if this result was due to plasmid loss, we performed a colony PCR of brain CFU using *ffluc* plasmid-specific primers (Fig. 4j and Supplementary Fig. 2). Only 1 out of 11 colonies tested retained the plasmid, indicating that GBS loses the plasmid *in vivo* over time, especially under conditions requiring extended analysis periods after infection. Overall, these data show that while the firefly luciferase-expressing GBS with the pLZ12Km2-P23R: TA: ffluc plasmid is a useful tool for models of infection leading to high organ colonisation, it is unsuitable for long-term studies due to plasmid instability over time *in vivo*.

### A stable luciferase reporter for *in vivo* imaging in GBS infections

Integration of the firefly luciferase gene into the bacterial chromosome ensures stability and transmissibility of the reporter. Thus, we next sought to insert the red-shifted firefly luciferase into the BM110 genome. We first amplified the *ffluc* gene from the pLZ12Km2-P23R: TA:*ffluc* vector, including the P23 promoter^29^, as well as two contiguous chromosomal GBS regions of 500 bp in a conserved intergenic region between the BQ8897_RS05445 and BQ8897_RS05450 genes, which encode a FAD-dependent oxidoreductase and L-lactate dehydrogenase, respectively (Supplementary Fig. 1). The three amplicons were combined and cloned into the pG1 integrative vector, with *ffluc* inserted into the middle of the 1 kb chromosomal sequence. Following GBS transformation, selection for homologous recombination at the targeted locus, derecombination, and vector loss, we isolated the *gluc*GBS-CC17 strain. Whole-genome sequencing of *gluc*GBS-CC17 confirmed the integration of *ffluc* at the targeted locus, with no remaining traces of the integrative vector and no other secondary mutations in the genome.

**Fig. 5:**
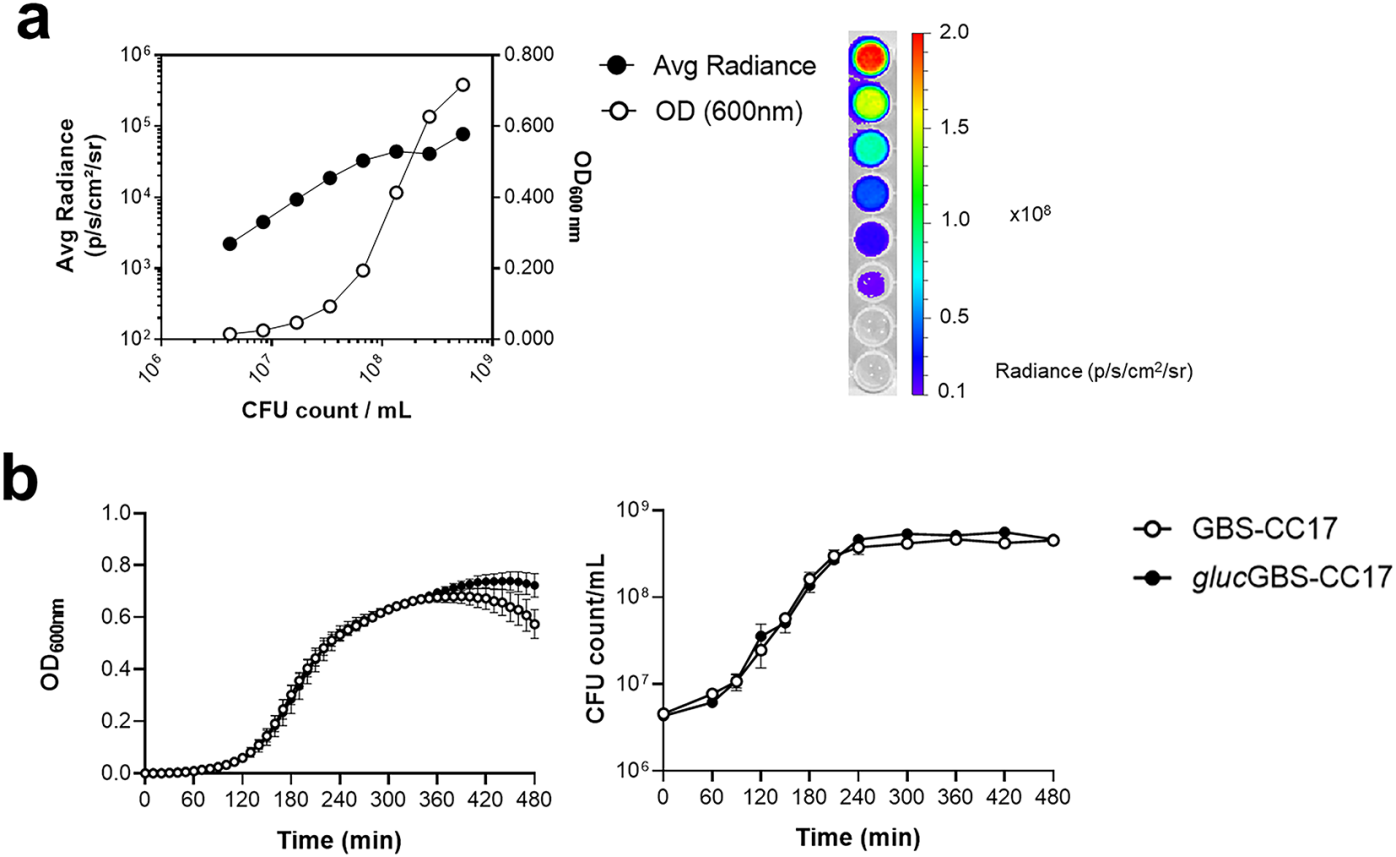
*In vitro* bioluminescence of the *gluc*GBS-CC17 with a chromosomally integrated luciferase. The red-shifted firefly luciferase gene was integrated into the genome of the WT strain to give the *gluc*GBS-CC17 strain. **(a)** Correlation of bioluminescent signal, optical density (OD) and CFU count. The *gluc*GBS-CC17 strain was grown until the mid-log phase and serially diluted in a 96-well plate. Left: Bioluminescence (Radiance, 0.5 mg/mL of Luciferin) and OD quantification on a plate reader (Synergy 2), and corresponding CFU counts. Field symbols represent radiance, whereas open symbols represent OD. Right: Bioluminescence signal imaged using IVIS Lumina LT. The detection of bioluminescent signals by the system resulted in the generation of signal maps overlaid on a grey-scale photograph of the 96-well plate. Bioluminescent signal plotted as radiance (photons/seconds/cm^2^/sr). Results represent data from 3 independent experiments. **(b)** Growth curves of WT and *gluc*GBS-CC17 strains. Optical density (left) measured every 10 min for 7 h on Synergy 2. GBS numbers counted every 30–60 min (right). Mean values ± SEM pooled from 3 independent experiments. Comparisons by two-way ANOVA.

**Fig. 6:**
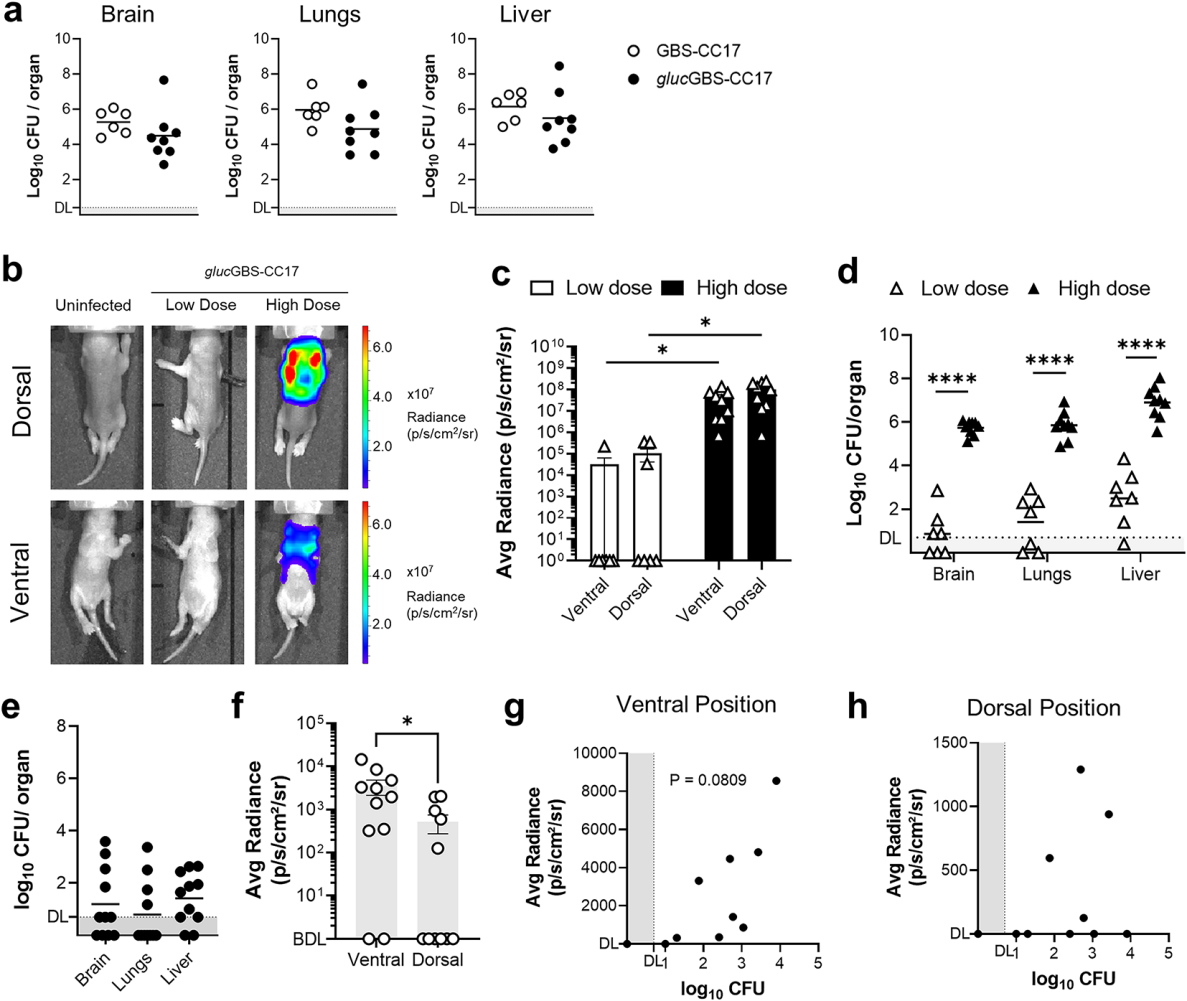
Infectivity and *in vivo* bioluminescence of *gluc*GBS-CC17. **(a)** Neonatal mice were inoculated intraperitoneally with 5 × 10^4^ CFU of *gluc*GBS-CC17. Bacterial load in the indicated organs at 18 hpi. Data as mean pooled from 2 independent experiments. Each symbol represents one pup (*n* = 6 WT; *n* = 8 *gluc*GBS-CC17). DL, detection limit = 0.7. **(b-d)** Neonatal mice were inoculated intraperitoneally with 5 × 10^4^ (low dose), 1 × 10^5^ (high dose) CFU of *gluc*GBS-CC17 or left uninfected. **(b)** Representative images of pups through the superimposition of the bioluminescence signal and a grey-scale photograph in the indicated positions. **(c)** The regions of interest (ROIs) were used to quantify the bioluminescence signal, expressed as average radiance (photons/s/cm^2^/steradian). Bars represent the mean ± SEM. Each symbol represents one animal (*n* = 6 Uninfected; *n* = 7 Low dose *n* = 9 High dose). **(d)** Bacterial load in the indicated organs and infection doses. Data as mean, each symbol represents one animal (*n* = 7 Low dose; *n* = 9 High dose). Statistical analysis using unpaired Student’s t-test. *P < 0.05, ****P < 0.0001. DL, detection limit = 0.4. **(e-h)** Pregnant mice were intra-vaginally inoculated with 5 × 10^4^ CFU of *gluc*GBS-CC17 during gestational. Three-day-old pups were analysed. **(e)** Bacterial load in the indicated organs. Data shown as mean pooled from 2 independent experiments. Each symbol represents one animal (*n* = 11). DL, detection limit = 0.7. **(f-h)** The ROIs were used to quantify the bioluminescence signal from the whole mouse, expressed as average radiance (photons/s/cm^2^/steradian). **(f)** Whole body average radiance in ventral and dorsal position. Mean ± SEM, each symbol represents one individual (*n* = 11). BDL, below detection limit. **(g-h)** Correlation between colonisation and the average radiance in the infected pups in the **(g)** ventral and **(h)** dorsal positions. Each symbol represents one individual (*n* = 10). BDL, below detection limit. *P < 0.05.

Similar to the previously developed reporter models, we started by assessing the *in vitro* luminescence signal and fitness. The optical density and CFU of *gluc*GBS-CC17 cultures reliably correlate with the radiance signal (Fig. 5a) and no differences in bacterial growth were found between the *gluc*GBS-CC17 and the WT strains (Fig. 5b). Next, we tested the infectivity and virulence *in vivo* using a haematological model of infection. No significant differences in organ colonisation 18 hpi was observed between the *gluc*GBS-CC17 and the WT strain (Fig. 6a), confirming that the integrated *ffluc* does not impact bacterial load in all tested organs. To test the *in vivo* bioluminescence, we infected mice using low (5 × 10^4^ CFU) and high (1 × 10^5^ CFU) inoculums of *gluc*GBS-CC17. The radiance values were significantly (*P* = 0.0292 ventral position, *P* = 0.0103 dorsal position) different and proportional to the infection doses (Fig. 6b-d). As expected, bacterial colonisation in the brain, lungs, and liver was significantly (*P* < 0.0001) higher in pups infected with the higher dose (Fig. 6d). These results indicate that this bioluminescent GBS strain is suitable for studying systemic infections. However, the *gluc*GBS-CC17 strain contains a single copy of the luciferase gene, resulting in a lower signal when compared to the *ffluc*GBS-CC17 strain containing the pLZ12Km2-P23R: TA:*ffluc* replicative vector (Figs. 4e and 6c).

The stable chromosomal integration of *ffluc* allows testing the *gluc*GBS-CC17 strain in our intra-vaginal (i.vag.) infection model of mother-to-pup transmission during delivery. At P3, the brain and liver of almost all pups were colonised, while only 4 out of 11 pups showed colonisation in the lungs (Fig. 6e). Despite the relatively low bacterial burden, significant luminescence signals were detectable in the pups (Fig. 6f). To further evaluate whether the radiance values could be correlated with bacterial brain colonisation a square region (ROIs) was drawn in the anatomical area of the brain and the bioluminescent signal obtained was plotted against the respective bacterial levels. No positive correlation was found regardless of ventral or dorsal mouse position (Supplementary Fig. 3a and b). However, close to statistical significance (*P* = 0.0809) was obtained when plotting the whole-body radiance obtained in the ventral position with the sum of bacterial colonisation in the lungs, liver, and brain (Fig. 6g), sustaining the validity of this strain to study moderate and severe GBS disease. Finally, aiming to improve sensitivity, we also tested a synthetic analogue of D-luciferin, cyclic alkylamino luciferin (CycLuc1), reported to produce a higher signal than D-luciferin^31,32^. Pups were i.p. infected with *gluc*GBS-CC17, and at 18 hpi different concentrations of CycLuc1 and time points were tested (Supplementary Fig. 4). However, no differences in the bioluminescent signal were observed compared to standard D-luciferin, regardless of concentration or time post-injection (Supplementary Fig. 4a and b). Altogether, our results show a bioluminescent GBS strain constitutively expressing the luciferase gene stably integrated in the chromosome suitable for studying GBS invasive disease.

## Discussion

Group B *Streptococcus* neonatal infections remain a major clinical problem. One limitation in studying GBS neonatal infection is that colonisation assessment entails animal euthanasia and, therefore, no longitudinal studies can be performed to study the kinetics of the infection, the disease progression, and the long-term sequelae. Here, we developed three bioluminescent GBS strains: one with the luciferase gene contained in the *lux* operon on a replicative vector, another with the *ffluc* gene also encoded on a replicative vector, and a third with the *ffluc* gene inserted into the GBS genome. Each bioluminescent strains are advantageous for specific applications: *in vitro* (*lux*GBS-CC17), *in vivo* for short term infections (*ffluc*GBS-CC17), or *in vivo* for complex infection models (*gluc*GBS-CC17). The choice between strains is based on the sensitivity (single or multi-copy) and the stability (vector-based or chromosomal integration) of the luciferase gene.

Our results suggest that the *lux*GBS-CC17 containing the *luxABCDE* operon on a replicative vector is appropriate for *in vitro* studies and not for invasive GBS infection models. *In vitro* luminescence is proportional to bacterial burden, consistent with results in other bioluminescent bacterial species^13^. The replicative vector is stable even in the absence of the antibiotic selective pressure, and no difference in growth with the WT strain is observed under standard conditions in TH broth. The main advantage is that this system does not require an exogenous substrate^13,26^. However, no luminescence signal was detected in infected animals, even when using a high infection dose. This unexpected result might be due to the bacteria spreading throughout the body, resulting in a dispersed, undetectable signal, or being located deeper within organs. The short wavelength (490 nm) of the lux operon limits tissue penetration^33,34^, especially due to absorption by hemoglobin^33,35,36^. Miller and colleagues^30^ showed that tissue penetration for a *Staphylococcus aureus* strain transformed with the same *lux* operon is limited up to 2 cm. Although 3-day-old C57BL/6 mice are thinner than 2 cm, their organ density is high due to their small size^37^, which could contribute to the limited detection. While it is unlikely that the P32 promoter is inactive during infection, and thus resulting in no bioluminescence signal, the optimisation of promoters should be explored in the future.

The second *ffluc*GBS-CC17 strain used contains the red-shifted firefly luciferase (*ffluc*) on a replicative vector, a *luc* reporter derivative validated for multiple streptococcal species^15^ overcoming the main limit of the *luxABCDE* system. The *ffluc*GBS-CC17 strain is therefore adapted to follow invasive infection in specific models. The *ffluc* has a longer wavelength (610 nm) than *lux* (490 nm)^18,33^, and improved bioluminescent signal and tissue penetration to detect bacteria in deeper tissues^18^. The replicative vector was previously used in GBS for *in vitro* bioluminescence analysis^15^. Here, we confirmed the *in vitro* stability of the replicative vector and validated the in vivo stability in GBS, as previously shown in *Streptococcus pyogenes*^23^. We also show that the pLZ12Km2-P23R: TA:*ffluc* vector did not impact on *in vitro* growth under standard conditions in TH broth and does not alter GBS virulence. The *ffluc*GBS-CC17 strain demonstrated robust bioluminescence, allowing visualisation and quantification of bacterial colonisation in the brain, lungs, and liver. The two infectious doses tested are the minimum for detecting luminescent signals associated with low and high bacterial loads, respectively. The lower dose of 5 × 10^4^ CFU is the minimum needed to visualize colonisation through bioluminescence, while doubling the dose to 10^5^ CFU produced higher, more uniform colonisation across all animals. Even when using a low inoculum to ensure increased pups’ survival, the BLI signal was proportional to the CFU colonisation in the organs, in both ventral and dorsal mice positions. Both radiance and CFU values were statistically significant between the lower and higher infection dose, confirming that the *ffluc*GBS-CC17 strain provides a reliable and sensitive method for tracking bacterial infection in live animals, even under non-selective antibiotic conditions.

The main limit of the *ffluc*GBS-CC17 strain emerged when tested in the vertical transmission model. Despite initial success in establishing infection, we observed plasmid loss over time, as evidenced by a lack of bioluminescent signal in later stages of infection. In this model, pregnant females are colonised in the vaginal mucosa at the gestational days 16 and 17, and allowed to deliver spontaneously^12^. During birth, the pups cross the vaginal canal and may inhale or ingest contaminated fluids, becoming infected^12^. As the pups are only studied after birth, the *ffluc*-containing plasmid should be stable for at least 3 to 7 days to detect luminescence during postnatal life. However, the stabilisation of the vector by the encoded toxin-antitoxin system is insufficient in vivo without an additional antibiotic selective pressure. Adding antibiotics to drinking water could stabilise the vector but would alter the microbiota. This plasmid instability under *in vivo* conditions suggests that while the *ffluc*GBS-CC17 strain is suitable for short-term studies and high-dose infections, it may not be ideal for long-term studies or infections acquired through natural routes.

The integration of the *ffluc* gene into the GBS genome resolves the stability issue. The *gluc*GBS-CC17 strain has a single copy of *ffluc* constitutively transcribed and integrated in a neutral intergenic region at the 3’ ends of two divergent transcribed genes. The genome of the *gluc*GBS-CC17 strain is identical to the genome of the WT parental strain, except for the integrated *ffluc* which does not affect bacterial growth and virulence. The stable integration ensures the maintenance of the reporter gene over extended periods in complex infection model of infection. When tested in the haematogenous model, *gluc*GBS-CC17 allowed to visualise and quantify bacterial colonisation of key organs such as the brain, lungs, and liver. The bioluminescent signal was proportional to CFU counts, providing a reliable measure of bacterial load. Importantly, the use of the *gluc*GBS-CC17 strain facilitated the detection of bioluminescence in the pups’ whole-body, even at lower infection doses.

The main limitation of the *gluc*GBS-CC17 strain is its weaker luminescence signals, linked to the presence of a single copy of the *ffluc* gene. This is especially limiting for brain colonisation in the vertical mouse model of infection. The low level of colonisation and the properties of the skull and blood-brain barrier (BBB), which impact and limit the access of D-luciferin to the parenchyma^31^, severely impacts the detection of the *gluc*GBS-CC17 strain. The luciferase substrate cyclic alkylamino luciferin (CycLuc1) has higher photon emission than D-luciferin at lower concentrations^31,32^ and increased BBB diffusion as it has increased lipophilicity^31,38^. Previous findings showed that CycLuc1 allows the detection of bioluminescence signals in cardiovascular brain regions, including the circumventricular subfornical organ (SFO) and paraventricular nucleus of the hypothalamus (PVN)^31^. Nevertheless, in our study CycLuc1 did not show a stronger luminescent signal regardless of the administered concentration or acquisition waiting time after injection. A possible explanation for this may be the lower bacterial load and the fact that it is widespread in the pup’s body, reducing the signal per area. While the introduction of CycLuc1 as a luciferase substrate initially showed promise for enhanced CNS imaging, our results highlight the complexities and limitations in achieving consistent bioluminescent signals in neonatal mice, necessitating further exploration of alternative strategies. Optimisation of the *ffluc* gene and promoter, or the use of alternative luminescence genes, could improve bioluminescent sensitivity to obtain a universal bioluminescent GBS strain.

In conclusion, we generated three GBS bioluminescent strains for pathogenic analysis, among which two containing the red-shifted firefly luciferase (*ffluc*) suitable to follow short-term and long-term invasive infections. This strain enables detailed tracking of bacterial colonisation and disease progression *in vivo*, providing better understanding of GBS pathophysiology. Moreover, it will pave the way for discovering novel approaches against neonatal GBS infections, while also allowing animal reduction and experiment refinement, implementing the ethical animal experiments 3R’s.

## Methods

### Bacterial strains, plasmids and growth conditions

*Enterococcus faecalis* containing the plasmid pSL101P32 carrying the *luxABCDE* cassette (Table 1) was kindly provided by Dzung B. Diep^27^. The pLZ12Km2-P23R: TA: fflucRT plasmid was a gift from Thomas Proft (Addgene, #88901)^18^. *E. faecalis* and *Escherichia coli* DH5α (Table 1) were grown in Brain Heart Infusion (BHI) broth or agar (Difco Laboratories) and Luria Bertani (LB) broth or agar (GRiSP, Lda), respectively, at 37 ºC. The wildtype GBS strain BM110, a CC-17 clinical isolate, was cultured at 37 ºC in Todd-Hewitt (TH) broth or agar (Difco Laboratories). For strains containing vectors (Table 1), antibiotics were added as appropriate at the following concentrations: spectinomycin (EMD Millipore) at 500 µg/mL (*E. faecalis*), 70 µg/mL (*E. coli*) or 200 µg/mL (GBS); kanamycin (Kan) (Sigma Aldrich) at 50 µg/mL (*E. coli*) or 200 µg/mL (GBS); erythromycin (Erm) (Sigma Aldrich) at 150 µg/mL (*E. coli*) or 5 µg/mL (GBS).

**Table 1:** Bacterial strains and plasmids nomenclature.

Bacterial strain	Plasmid	Selective antibiotic	Nomenclature (if applicable)
*Enterococcus faecalis*	PSL101P32	Spectinomycin	
*Escherichia coli* DH5α	PSL101P32	Spectinomycin	
*Escherichia coli*	pLZ12Km2-P23R: TA: fflucRT	Kanamycin	
*Escherichia coli* XL-1 blue	pG-oxi-luc-ldh	Erythromycin	-
Group B *Streptococcus* BM110		-	GBS-CC17
Group B *Streptococcus* BM110	PSL101P32	Spectinomycin	*lux*GBS-CC17
Group B *Streptococcus* BM110	pLZ12Km2-P23R: TA: fflucRT	Kanamycin	*ffluc*GBS-CC17

### Bacterial growth and *in vitro* luminescence

*Lux*GBS-CC17 was grown overnight (ON) and 1:100 subcultured in non-vented culture flasks in 30 mL of TH with 5 µg/mL erythromycin at 37 ºC, for 7 h. The optical density (OD) at 600 nm (OD_600 nm_) was measured every 30–60 min using a spectrophotometer (Biochrom Libra S6+). The culture was plated at the same timepoints on TH agar with 5 µg/mL erythromycin and incubated at 37 ºC ON for CFU count. All the other strains were 1:100 subcultured in TH with the respective antibiotic, in transparent 96-well microplates. The cultures were maintained at 37 ºC and measurements were performed every 10 min for 7 h, on Synergy 2 Plate reader (OD_600 nm_).

For the luminescent signal, the strains were grown in non-vented culture flasks containing 30 mL of TH. An aliquot of the culture was taken at indicated timepoints and read on Synergy 2 Plate reader, using a white 96-well flat bottom microplate (settings at 37 ºC, 3s shaking before reading and luminescence read with 150 of gain). In all experiments technical duplicates and biological triplicates were performed.

## GBS transformation

Plasmids were extracted using commercial kits according to manufacturer’s instructions. The pSL101P32 or pLZ12Km2-P23R: TA: fflucRT vectors were introduced in GBS by electroporation. Electrocompetent bacteria were prepared starting from an ON culture in TH at 37 °C used to inoculate (1:10) fresh medium. The culture was grown at 37 °C until exponential growth phase (OD 600 nm around 0.9). Then, one volume of a 1 M sucrose solution and 0.05% of glycine were added, and the culture was incubated for 1 h at 37 ºC with mild shaking. The culture was centrifuged at 2,000 *g* for 10 min at 4 °C, washed twice with electroporation buffer (7 mM sodium phosphate, 500 mM saccharose, 1 mM magnesium chloride, pH 7.4), and re-suspended in electroporation buffer with 15% glycerol. The culture was stored at -80 °C until electroporation was performed. For electroporation, 6 µL of plasmid was added to 100 µL of competent bacteria, and the mixture was electroporated at 200 Ω, 2500 V / cm electric field, 25 µF. Thereafter, 900 µL of TH was immediately added and the solution was incubated for 1 h 30 min, at 37 °C. The bacteria were then plated on TH plates containing spectinomycin or kanamycin at 37 °C. Isolated GBS spectinomycin-resistant transformant containing the vector pSL101P32 was referred to as *lux*GBS-C17 and the kanamycin-resistant transformant containing the vector pLZ12Km2-P23R: TA: fflucRT as *ffluc*GBS-CC17.

## Polymerase chain reaction (PCR)

Colony PCR was performed using the following primers (Table 2) to target the *lux*ABCDE operon: *LuxA* sense (5’GCCTACCGATGATATTAAGTTG 3’) and *LuxB* anti-sense, *LuxD* sense and *LuxE* antisense; and the following primers to target *Ffluc* gene: *Ffluc* sense and antisense. The PCR reaction comprising NZYTaq 2x Green Master Mix (NZYTech) was run under the following conditions: an initial denaturation step at 95 °C for 5 min, 30 cycles of 95 °C for 30 s, 55 °C for 30 s and 72 °C for 25 s, followed by a final extension step at 72 °C for 10 min. The PCR products were run in a 1% agarose gel, containing Green Safe Premium (NZYTech). Gel images were acquired in a gel Imaging system (Gel-Doc XR+, Bio-Rad) using Image Lab (V6.0.1, Bio-Rad) software.

**Table 2:** Oligonucleotide sequences of the utilised primers.

Primer ID	Oligonucleotide sequence
*LuxA* (sense)	GCCTACCGATGATATTAAGTTG
*LuxB* (anti-sense)	ACCACACTAATGGATCGCTCG
*LuxD* (sense)	TGCGCGGATAGCTTATGCAA
*LuxE* (anti-sense)	ACCACACTAATGGATCGCTCG
*Ffluc* (sense)	AAGGTCCTGCCCCATTTTACC
*Ffluc* (anti-sense)	GCGGCAAATGACTGGTTACG
879	GAATTCGTAATCATGTCATAGCTGT
880	GAATTCGTAATCATGTCATAGCTGT
1688	CCTGCAGGTCGACTCTAGAGGATCCGACAATTTGGGATAATTCTCGTG
1689	GAGCTTAAGAATTGCCGCTCTAGATCATCCTTTAACAAGGTCAAAAATG
1690	GTTAAGCGAAATGCTTAGCCTTTTACGAAAAGCCCTGACAACCCTTGTTC
1691	CATTTTTGACCTTGTTAAAGGATGATCTAGAGCGGCAATTCTTAAGCTCG
1692	CGAGCTTAAGAATTGCCGCTCTAGATCATCCTTTAACAAGGTCAAAAATG
1694	GTTGATATGGTGATCTTAGCTGTTG
1695	GTTATTGGTTCAGGTACTTCACTTG

### Plasmid stability

GBS strains containing the luminescent plasmid (pSL101P32 or pLZ12Km2-P23R: TA: fflucRT) were grown in TH ON at 37 ºC, with or without antibiotics and subsequently subcultured 1:1000 in fresh TH, with or without antibiotics, and incubated at 37 °C for 24 h. This step was repeated every 24 h for 8–10 days. Subcultures were serially diluted and plated on TH agar medium, with or without selection, every day and incubated ON at 37 ºC. Plasmid stability was assessed given the difference in the CFU count in the presence and absence of antibiotic selection.

## Construction of pG-oxi-luc-ldh

To construct the *gluc*GBS-CC17 strain with a chromosomal *ffluc* gene, we constructed the pG-oxi-luc-ldh integrative vector (Supplementary Fig. 5). First, the the *ffluc* gene was amplified from the pLZ12Km2-P23R: TA:*ffluc* vector using high-fidelity DNA polymerase (Phusion, New England Biolabs) and primers 1690 and 1691 (Table 2).Two contiguous 500 bp region of the BM110 genome were similarly amplified using primers 1688 and 1689 and 1692 and 1695 (Table 2). The three amplicons contain overlapping sequences and were purified (QIAquick PCR purification, Qiagen) and mixed for a final PCR with the external primers 1688 and 1693 (Table 2). The final amplicon was gel purified (QIAquick gel purification, Qiagen) and used for Gibson assembly into the pG1 thermosensitive vector previously amplified with the 879 and 880 primers (Table 2).

Integration of *ffluc* into the GBS CC17 genome.

The pG-oxi-luc-ldh vector was introduced in *E. coli* XL-1 blue (Stratagene) and selected with erythromycin. After purification and sanger sequencing (Eurofins Genomics), the pG-oxi-luc-ldh was introduced in BM110 by electroporation with erythromycin selection at 30°C, the permissive temperature for vector replication.Chromosomal integration at the targeted locus was selected by growing isolated transformants at 37°C on THY agar in the presence of erythromycin. Following a second round of isolation on THY with erythromycin, a single colony was inoculated into 10 ml of THY without antibiotics, grown at 30°C, and sub-cultured four times under the same conditions. Diluted cultures (d = 10^− 5^) were then spread on THY agar, grown overnight at 37°C, and single colonies (n = 48) were inoculated into 150 µl of THY in microwell plates. After 4 hours of incubation at 37°C, replicas were made using a 96-pin microplate replicator (Boekel) on THY with and without erythromycin to differentiate colonies that retained or lost the vector. Erythromycin-sensitive colonies were then tested by PCR with primers 1694 and 1695 (Table 2) to determine whether they had restored a wild-type genomic sequence (indicating loss of the integrated lux gene) or whether the lux gene had precisely integrated at the targeted genomic position. The outcome (WT back or *lux* integration) depends on the side of homologous recombination (5’ or 3’) between the flanking genomic regions cloned around the *lux* gene. Colonies with the integrated *lux* gene were isolated and their genomic DNA were purified (DNeasy Blood and Tissue kit, Qiagen) and sequenced (Illumina, Novogen). High-quality reads were mapped against the BM110 genome (362x genome coverage) and analyzed for SNP, indel, and genomic rearrangement (Genious Prime, Biomatters Ltd). The selected *gluc*GBS-CC17 strain, with *lux* inserted at position 1,000,921 in the BM110 genome (NCBI RefSeq NZ_LT714196) and without any secondary mutation, was then stored in 20% glycerol at -80 °C.

## Adhesion and invasion assays

The human colonic epithelial cell line Caco-2^12^, derived from a human colorectal adenocarcinoma (ATCC), was cultured at 37 ˚C in a 5% CO_2_ atmosphere in DMEM high glucose with GlutaMax (HyClone) supplemented with 10% heat-inactivated FBS, 1% sodium pyruvate (Gibco) and 1% penicillin/streptomycin ( 6.0 µg/ mL / 10.0 µg/mL, Biowest). The cell line was grown until cells reached 80% confluence. For infection assays, cells were seeded on 12 well-standard plates at 3 × 10^5^ cells/well for 4 days at 37 °C under a 5% CO_2_ atmosphere. Briefly, cells were washed thrice and infected with GBS in media without FBS or antibiotics at a multiplicity of infection (MOI) of 30. The number of adhered cells was counted to adjust the bacterial numbers. Infection was allowed for 3 h, followed by five washes to remove nonadherent bacteria. Subsequently, the cells were lysed with TrypLE Express (Gibco) containing 0.2% Triton X-100 for 10 min at 37 ºC. The bacterial adhesion was quantified by subtracting the intracellular bacteria from the total cell-associated (intracellular plus surface-adherent bacteria).

For invasion assays, 3-hour-infected cell monolayers were further incubated with cellular culture media containing 100 µg/mL gentamycin (Lonza) for 1.5 h to kill the extracellular bacteria, before cell lysis. Appropriate dilutions were plated on TH agar plates, and CFU were counted. The percentage of invasion was calculated relative to the bacterial inoculum.

### Animal handling and ethics statement

C57BL/6 mice were purchased from Charles River Laboratories and housed at the Instituto de Investigação e Inovação em Saúde (i3S) animal facility under a 12 h light/dark cycle and *ad libitum* access to food and water. All animal procedures were approved by the Ethics Committee of the i3S, and the Portuguese national authority the Direção Geral de Alimentação e Veterinária (DGAV). All animal experiments were performed in accordance with the recommendations of the European Convention for the Protection of Vertebrate Animals used for Experimental and Other Scientific Purposes (ETS 123) and Directive 2010/63/EU and Portuguese rules (DL 113/2013). All animal experiments were planned to minimise mice suffering and number, and always handled by licensed investigators. Additionally, all experiments were performed in accordance with relevant guidelines and regulations, including the ARRIVE guidelines for animal experiments.

### *In vivo* mouse infection models

Overnight cultures of the different GBS strains were subcultured 1:100 on TH broth and grown until mid-log phase (OD_600nm_ around 1.000) at 37 °C. Bacteria were washed twice with PBS, and the OD_600nm_ was adjusted to ≈ 0.600 ± 0.005, corresponding approximately to 2.0 × 10^8^ CFU / mL. Inocula were prepared by further dilution from this bacterial suspension.

Two-day-old C57BL/6 mice were intraperitoneally (i.p.) infected with the indicated CFU of WT GBS or luciferase-expressing strains in 40 µL of total volume. Survival curves were determined over a 36 h experimental period. To assess bacterial colonisation, mice were euthanised by decapitation following isoflurane anaesthesia. The brain, lungs and liver were aseptically removed 18 h post-infection, homogenised, serially diluted in PBS, and plated on TH agar medium with or without antibiotic selection for CFU counts.

To vertically infect neonatal mice, we adapted our previously described mouse model of infection^12^. Pregnant C57BL/6 mice were intravaginallyinoculated, using a micropipette, with 40 µL of GBS suspension containing 3 × 10^4^ CFU, at the gestation (G) days 16 and 17. The presence of a vaginal plug was considered the day 1 of gestation. Females were allowed to deliver spontaneously, and infected pups were kept with their mothers throughout the experimental time. Organs were collected and plated as previously described.

### Bioluminescence imaging

For the *in* *vitro* correlation between the bioluminescence signal of luciferase-expressing GBS strains, the CFU and the OD, bacteria were grown as described above and serially diluted 1:2 in white opaque flat clear bottom or transparent flat bottom 96-well plates. The luminescence and the absorbance at 600 nm were quantified on Synergy 2 (BioTek). For *ffluc*GBS BM110, 1:1 D-Luciferin potassium salt (0.5 mg / mL, Perkin Elmer) was added to the wells 5 min before luminescence quantification. Bioluminescence imaging was assessed on the IVIS Lumina LT system (Perkin Elmer). According to the strain, bacterial dilutions were plated on TH agar, with or without selection, for CFU enumeration.

For *in* *vivo* studies, C57BL/6 mice infected with the different bioluminescent strains were anaesthetised with 2.5% isoflurane (O_2_ flow of 1.0 L/minutes). In the case of mice infected with *ffluc*GBS, 20 µL of the indicated concentrations of D-Luciferin or CycLuc1 sodium salt (AOBIOUS, AOB6377) were administered s.c. The substrate was allowed to be distributed in the body of the anesthetised animal for 5 min, unless stated otherwise, before image acquisition. Pups were allowed to recover from the anaesthesia before returning to the respective cage. Bioluminescence imaging was acquired on the IVIS Lumina LT system (Perkin Elmer) or Newton 7.0 (Vilber).

### Statistical analysis

All data were analysed and graphically represented using GraphPad Prism software (v.9.0.1, GraphPad Software Inc. CA). Means and standard errors of the means (SEM) were calculated and correspond to the indicated independent experiments. Student’s t-test was used to analyse differences between two experimental groups. The differences between multiple groups were performed by one-way or two-way ANOVA, whenever appropriate, with Tukey’s Multiple Comparison Test. The normality of the data was verified by the Shapiro-Wilk normality test. Survival curves were plotted using Kaplan-Meier plot, and statistical significance was determined using the log-rank (Mantel-Cox) test. CFU data were log_10_ transformed. Differences were considered significant for *P* ≤ 0.05 and represented by *P < 0.05; **P < 0.01; ***P < 0.001; ****P < 0.0001. The number of samples and independent experiments is expressed in the respective legend.

## Electronic supplementary material

Below is the link to the electronic supplementary material.

Supplementary Material

## Data Availability

All data generated or analysed during this study are included in this published article (and its Supplementary Information files). Further inquiries can be directed to the corresponding author, Elva Bonifácio Andrade.
