# Supplementary material for: Development of bioluminescent Group B streptococcal strains for longitudinal infection studies
Source: Sci Rep. 2024 Oct 18;14:24439. doi: 10.1038/s41598-024-74346-z (PMC11489706; doi:10.1038/s41598-024-74346-z)
Supplement: Supplementary file 1 — Supplementary Material [file 41598_2024_74346_MOESM1_ESM.pdf]

Supplementary Figures

Development of bioluminescent Group B *streptococcal* strains for longitudinal infection studies

Inês Lorga[1,2], Rafaela Geraldo[1,3], Joana Soares[2], Liliana Oliveira[2], Arnaud Firon[4] and Elva Bonifácio Andrade[1,2,5*]

Supplementary Figure 1

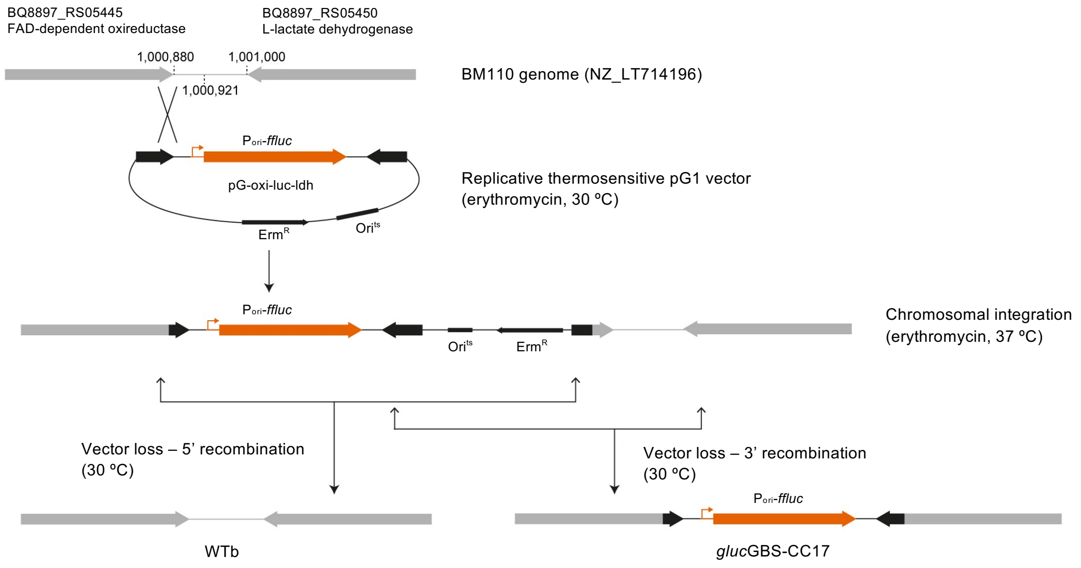

Supplementary figure 1. Insertion of the *ffluc* gene into the genome of BM110. The pG-oxi-luc-ldh integrative vector was constructed by the insertion of the *ffluc* gene (orange) flanked by 500 bp of chromosome sequences (large black arrows) into the pG1 vector. The pG-oxi-luc-ldh vector was introduced in GBS BM110 by electroporation with erythromycin selection at 30 °C, the permissive temperature for vector replication. Chromosomal integration by single crossing-over at the targeted locus (black cross) was selected by growing isolated transformants at 37 °C in the presence of erythromycin. De-recombination and vector loss were selected at 30 °C without antibiotic and can lead to the restoration of a WT sequence (left: single crossing-over between homologous 5' sequences) or to the precise integration of the fflux gene into the chromosome (of the vector (right: single crossing-over between homologous 3' sequences). For simplicity, only homologous recombination at the 5' end of P_{ori}-*fflux* is shown for the chromosomal integration step, although single crossing-over can also occur at the 3' end.

Supplementary Figure 2

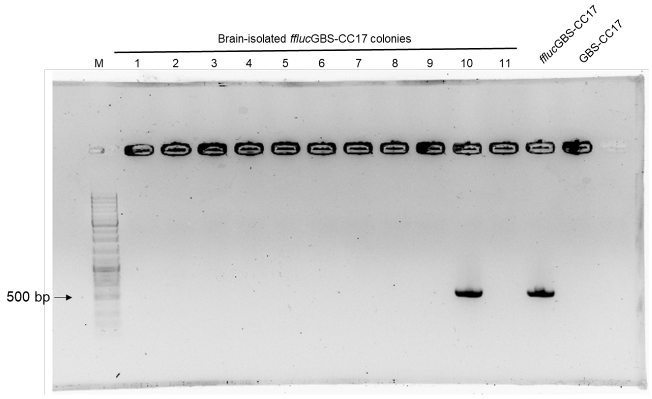

Supplementary figure 2. Colony PCR of brain-isolated *ffluc*GBS-CC17. Pregnant mice were intra-vaginally inoculated with $8x10^4$ *ffluc*GBS-CC17. Three-day-old pups were analysed. Agarose gel showing colony PCR for screening *ffluc*GBS-CC17 collected from pups' brains, using plasmid-targeted primers (495 bp amplicon). M, molecular weight marker (10 kb GeneRuler DNA ladder mix); 1-11, bacteria isolates; *ffluc*GBS-CC17 and WT GBS-CC17 as positive and negative controls, respectively.

Supplementary Figure 3

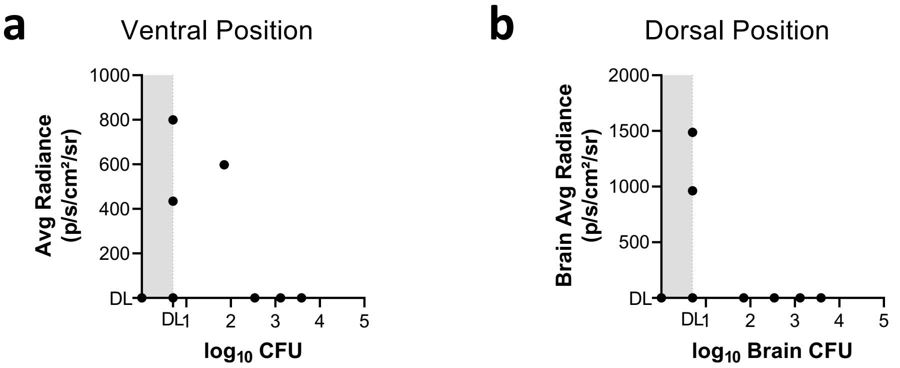

Supplementary figure 3. Evaluation of the brain bioluminescent signal in the vertical model of infection with the *gluc*GBS-CC17. Pregnant C57Bl/6 mice were intra-vaginally inoculated with $5x10^4$ CFU of *gluc*GBS-CC17 at gestational days 16 and 17. Three-day-old pups were analysed. **(a-b)** The ROIs were used to quantify the bioluminescence signal from the mouse head, expressed as average radiance (photons/s/cm^2/steradian). Correlation between the average radiance in the infected pups' head and the brain colonisation, in the **(a)** ventral and **(b)** dorsal positions. Each symbol represents one individual (n = 8).

Supplementary Figure 4

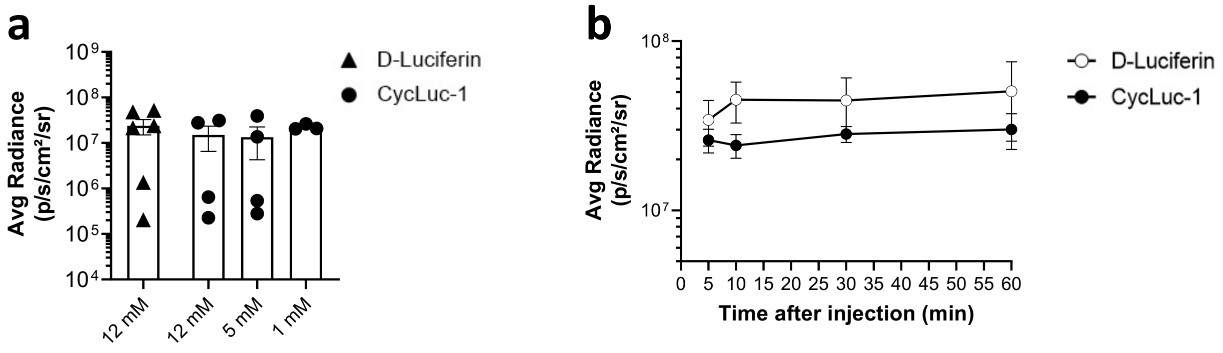

Supplementary figure 4. Evaluation of the synthetic luciferase CycLuc1. Two-day-old C57BL/6 pups were infected intraperitoneally with 8x10^4 CFU of the bioluminescent strain (*gluc*GBS-CC17) or left uninfected. Analyses were performed 18 hours post infection **(a)** Average radiance (photons/s/cm^2/steradian) of the whole body of infected pups. Data shown as mean ± SEM are represented in bars pooled from 2 independent experiments. Each symbol represents one animal (n = 3 for 1mM CycLuc; n = 4 for the remaining conditions). **(b)** Kinetics of the average radiance (photons/s/cm^2/steradian) of the whole body of pups infected with the *gluc*GBS, injected with D-Luciferin or CycLuc-1. Data shown as mean ± SEM pooled from 3 independent experiments (n = 6 D-Luciferin; n = 8 CycLuc-1).

Supplementary Figure 5

pG1….CGACAATTTGGGATAATTCTCGTGATGATATCAATTATATTGCTCTTGCTTCAAACGCCGTTCGTACTGG
TATTGTAGCAGCTCACAATGCATGTGGTACAGAGCTTGAAGGTGCTGGTGTCCAAGGGTCAAATGGTATCTCA
ATCTATGGCCTTAACATGGTGTCAACTGGTTTGACTTTAGAAAAAGCAAAACAAGCTGGGTACAATGCAGTTG
AAACAGGCTTTAATGACCTTCAAAAACCAGAATTTATCAAACACAATAATCATGAAGTAGCTATTAAGATTGTC
TATGATAAAGATAGCCGAGTTATCCTTGGTTGCCAAATGGTCTCACACGAAGATGTATCTATGGGAATTCATAT
GTTCTCACTTGCTATTCAAGAAAAAGTTACTATTGAAAAGCTTGCTTTGACCGATATCTTCTTCTTACCACATTT
TAATAAGCCATATAACTATATTACAATGGCTGCATTAGGCGCTAAAGATTAATTTAAAATTAAAAAGGTTAAGCG
AAATGCTTAGCCTTTTACGAAAAGCCCTGACAACCCTTGTTCCTAAAAAGGAATAAGCGTTCGGTCAGTAAAT
AATAGAAATAAAAAATCAGACCTAAGACTGATGACAAAAAGAGAAAATTTTGATAAAATAGTCTTAGAATTAA
ATTAAAAAGGGAGGCCAAATATAATGAAAAATATGAATGACAATGATGTTCCGCGGTGGCGGCAATTCGGAG
GATATATATGGAAGATGCTAAGAATATCAAGAAAGGTCCTGCCCCATTTTACCCACTTGAGGATGGTACTGCTG
GGGAACAATTGCATAAGGCTATGAAACGTTACGCCCTAGTTCCAGGTACTATCGCTTTTACAGATGCTCATATT
GAGGTGAATATTACATATGCGGAATATTTTGAAATGAGCGTTCGCCTTGCGGAAGCTATGAAGCGTTACGGAC
TAAATACTAATCATCGAATCGTTGTTTGCAGTGAAAACTCTTTGCAGTTCTTTATGCCTGTCTTAGGTGCACTTT
TTATTGGAGTAGCAGTTGCTCCTGCAAATGATATTTACAATGAACGAGAATTACTGAATAGCATGAACATTTCT
CAACCAACTGTAGTGTTTGTTTCTAAGAAAGGATTACAGAAAATCTTGAATGTGCAGAAAAAGCTACCAATAA
TTCAGAAGATCATTATTATGGACAGCAAGACTGATTACCAGGGTTTCCAATCAATGTACACTTTCGTAACCAGT
CATTTGCCGCCTGGCTTCAATGAGTACGACTTTGTCCCAGAATCTTTTGATCGAGATAAGACAATTGCGTTAAT
TATGAATAGCTCAGGTTCTACTGGTCTACCTAAAGGCGTGGCTTTGCCTCACCGTGCTTTATGCGTACGCTTTT
CTCACGCTCGTGATCCTATTTTTGGAAATCAGATTGCTCCAGATACAGCGATTTTATCTGTTGTGCCTTTCCATC
ATGGCTTTGGAATGTTTACAACTTTGGGCTACCTCATCTGTGGCTTTAGGGTTGTGCTAATGTATCGCTTTGAG
GAAGAATTATTTCTACGATCCTTACAAGACTATAAGATACAGACGGCACTTTTAGTTCCAACGTTATTTAGTTTT
CTCGCAAAAAGTACTCTAATCGATAAATATGATTTATCTAACTTGCATGAGATAGCTTCTGGAGGCGCACCATT
GTCAAAAGAAGTCGGTGAGGCTGTTGCAAAAAGATTTCATTTACCAGGCATTCGTCAAGGGTACGGTCTAAC
GGAAACAACATCGGCTATTCTTATTACACCAAAAGGTGATGATAAGCCTGGGGCTGTTGGAAAAGTTGTTCCT
TTCTTCGAAGCAAAGGTAGTGGATCTTGATACTGGAAAAACATTAGGCGTTAACCAAAGAGGCGAGCTGTGT
GTCAGAGGCCCTATGATTATGAGTGGCTACGTTAATAATCCAGAGGCTACGAACGCATTAATTGATAAAGATG
GATGGTTGCATTCAGGTGACCTTGCTTATTGGGATGAGGATGAACACTTCTTTATAGTAGGACGACTAAAATC
TCTCATCAAGTATAAAGGATATCAAGTCGCCCCAGCTGAATTGGAGTCAATTCTTTTACAACATCCTAATATCTT
TGACGCTGGAGTCGCTGGTTTGCCTGACGATGATGCGGGTGAACTCCCGGCTGCTGTGGTCGTGCTTGAGC
ATGGAAAAACCATGACAGAGAAAGAAATTGTGGATTACGTAGCCTCACAAGTTACTACCGCTAAGAAATTAC
GTGGTGGTGTAGTTTTCGTCGATGAAGTTCCTAAAGGATTAACTGGTAAACGTGACGCTCGTAAGATTCGTGA
AATTTTGATAAAGGCGAAAAAGGGTGGCAAAAGCAAACTGTTAATTAAGGATCCTCTAGAGGTACCGTCGAC
AAAAGGGGCCCGAGCTTAAGAATTGCCGCTCTAGATCATCCTTTAACAAGGTCAAAAATGTAACGGCAAAGA
AAAAGCTAAGATATTCCTATCCTAGCTTTTTTTCAACTATGTTAGTTTTTTGATGCTTCTTGGAATTCTGGGTTTT
TCCAAGCTTCGTCAATAATATCTTTTAATTGTTCAGCTGAGGCTTGCATTTTTTGAAGTTCAGCATCATTTAATG
GGATATTAACTGGACGAACGATACCATGTGCACCTACGATTGCAGGCTGACCGATAAAGACATCTTTAACATC
ACCGTATTGGCCTTCTTGATATACAGATAATGGAAGAACTGCATTTTCATCATCAAGGATAGCTTTAGTAATACG
AGCAAGTGCGACAGCAATACCGTAATATGTAGCACCTTTTTTGTTGATGATTGAGTATGCAGCGTCACGAACT
GAAATAAACAAATCAACAAGTCCTTGTTCATCAATATCGCGATTTTCTTGTAACCATTGTTCAAGTTGGACACC
AGCAACGTTAGCATGTGACCAAACGGCAAATTCTGAGTCACCGTGTTCAC…pG1

Supplementary figure 5. Complete sequence of the pG-oxi-luc-ldh insert. The nucleotide sequence of the insert cloned into pG that forms the pG-oxi-luc-ldh integrative vector. Letters in Yellow, P23 promoter; Red, ffluc; Light green, 3' end of the chromosomal FAD-dependent oxidoreductase encoding GBS gene (NCBI RefSeq BQ8897_RS05445); Dark green, 3' end of the chromosomal L-lactate dehydrogenase encoding GBS gene (NCBI RefSeq BQ8897_RS05450). Underlined letters, annealing sequences for primers used to construct pG-oxi-luc-ldh.
